# NUP85 as a Neurodevelopmental Gene: From Podocyte to Neuron

**DOI:** 10.3390/genes14122143

**Published:** 2023-11-27

**Authors:** Antonella Gambadauro, Giuseppe Donato Mangano, Karol Galletta, Francesca Granata, Antonella Riva, Laura Massella, Isabella Guzzo, Giovanni Farello, Giovanna Scorrano, Ludovica Di Francesco, Giulio Di Donato, Carolina Ianni, Armando Di Ludovico, Saverio La Bella, Pasquale Striano, Stephanie Efthymiou, Henry Houlden, Rosaria Nardello, Roberto Chimenz

**Affiliations:** 1 Department of Human Pathology in Adult and Developmental Age “Gaetano Barresi”, University of Messina, Via Consolare Valeria 1, 98124 Messina, Italy; gambadauroa92@gmail.com (A.G.); roberto.chimenz@polime.it (R.C.); 2 Department of Biomedicine, Neuroscience and Advanced Diagnostics, University of Palermo, 90127 Palermo, Italy; giuseppedonato.mangano@unipa.it; 3 Department of Biomedical, Dental Science and Morphological and Functional Images, Neuroradiology Unit, University of Messina, Via Consolare Valeria 1, 98125 Messina, Italy; k.galletta@unime.it (K.G.); francesca.granata@unime.it (F.G.); 4 Unit of Medical Genetics, IRCSS Giannina Gaslini Institute, Via Gerolamo Gaslini 5, 16147 Genoa, Italy; antonella.riva@edu.unige.it (A.R.); strianop@gmail.com (P.S.); 5 Department of Neurosciences, Rehabilitation, Ophthalmology, Genetics, Maternal and Child Health, University of Genoa, Via Gerolamo Gaslini 5, 16147 Genoa, Italy; 6 Division of Nephrology, Department of Pediatric Subspecialties, Bambino Gesù Children’s Hospital, Istituto di Ricovero e Cura a Carattere Scientifico (IRCCS), 00165 Rome, Italy; laura.massella@opbg.net (L.M.); isabella.guzzo@opbg.net (I.G.); 7 Department of Pediatrics, University of L’Aquila, 67100 L’Aquila, Italy; giovanni.farello@univaq.it (G.F.); giovanna.scorrano@gmail.com (G.S.); difrancesco.ludovica@gmail.com (L.D.F.); giuliodidonato890@gmail.com (G.D.D.); carolina.ianni@gmail.com (C.I.); armandodl@outlook.com (A.D.L.);; 8 Department of Neuromuscular Disease, UCL Queen Square Institute of Neurology and The National Hospital for Neurology and Neurosurgery, London WC1N 3BG, UK; s.efthymiou@ucl.ac.uk (S.E.); h.houden@ucl.ac.uk (H.H.)

**Keywords:** *NUP85*, steroid-resistant nephrotic syndrome, nephrotic syndrome type 17, epileptic spasm, developmental delay, microcephaly

## Abstract

Pathogenic gene variants encoding nuclear pore complex (NPC) proteins were previously implicated in the pathogenesis of steroid-resistant nephrotic syndrome (SRNS). The *NUP85* gene, encoding nucleoporin, is related to a very rare form of SRNS with limited genotype–phenotype information. We identified an Italian boy affected with an SRNS associated with severe neurodevelopmental impairment characterized by microcephaly, axial hypotonia, lack of achievement of motor milestones, and refractory seizures with an associated hypsarrhythmic pattern on electroencephalography. Brain magnetic resonance imaging (MRI) showed hypoplasia of the corpus callosum and a simplified gyration of the cerebral cortex. Since the age of 3 years, the boy was followed up at our Pediatric Nephrology Department for an SRNS, with a focal segmental glomerulosclerosis at renal biopsy. The boy died 32 months after SRNS onset, and a Whole-Exome Sequencing analysis revealed a novel compound heterozygous variant in *NUP85* (NM_024844.5): 611T>A (p.Val204Glu), c.1904T>G (p.Leu635Arg), inherited from the father and mother, respectively. We delineated the clinical phenotypes of NUP85-related disorders, reviewed the affected individuals so far reported in the literature, and overall expanded both the phenotypic and the molecular spectrum associated with this ultra-rare genetic condition. Our study suggests a potential occurrence of severe neurological phenotypes as part of the *NUP85*-related clinical spectrum and highlights an important involvement of nucleoporin in brain developmental processes and neurological function.

## 1. Introduction

Nephrotic syndrome (NS) is defined by the presence of (i) severe proteinuria (≥40 mg/m^2^/h or a urine protein/creatinine ratio of ≥200 mg/g or 3+ proteins on urine dipstick), (ii) hypoalbuminemia (albumin < 2.5 g/dL), and (iii) edema [1]. Most children and adolescents with idiopathic NS show remission within 4 weeks of prednisone or prednisolone treatment at 60 mg/m^2^/day (or 2 mg/kg/day, maximum 60 mg/day). A complete remission is defined as a urine protein/creatinine ratio of <200 mg/mL or negative or trace dipstick on ≥3 consecutive occasions [2]. Patients with a lack of complete remission within 8 weeks of treatment with prednisone or prednisolone at standard doses are considered to have steroid-resistant nephrotic syndrome (SRNS) [2,3]. Steroid resistance is an important factor in the future risk of end-stage renal disease. The expansion of renal and neurodevelopmental molecular mechanisms is aided by the growing application of Next-Generation Sequencing (NGS), and an increasing number of phenotypes with neurological involvement are correlating with a progressively precise genotypic characterization [4,5,6,7,8]. SRNS can have a genetic basis in some patients, and pathogenic variants in more than 70 genes have been recognized as the cause of the illness so far. Genes associated with SRNS can encode different critical components of the slit diaphragm (NPHS1, NPHS2, CD2AP, and PLCE1) or the cytoskeleton (ACTN4, MHY9, and IFN2). More recently, genes encoding glomerular basement membrane/matrix proteins (*LAMB2*, *ITGA3*, and *COL4A3-5*), mitochondrial proteins (*COQ2*, *COQ6*, and *ADCK4*), nuclear proteins (*WT1*, *LMX1B*, *NUP93*, *NUP107*, *NUP205*, and *SMARCLA1*), and other intracellular proteins (*TRPC6*, *SCARB2*, *APOL1*, *DGKE*, *CUBN*, and *GAPVD1*) have also been identified in association with SRNS. However, there is a great genetic and clinical heterogeneity, and individual mutations can be responsible for distinct phenotypes and different natural histories of the disease [3]. Nucleoporins (NUPs) are structural units of nuclear pore complexes (NPCs) that assemble during cell proliferation into the nuclear envelope, where they contribute to fusing the outer and inner nuclear membranes, which are important in glomerular function. The NPCs form a selective barrier between the cytoplasm and the nucleoplasm, facilitating the bi-directional diffusion of small molecules (up to 9 nm) and active transport of macromolecules (up to about 40 nm), such as the nuclear import of proteins, the export of RNA and ribonucleoproteins, and the bi-directional shuttling of molecules involved in signaling and biogenesis [9,10].

The rupture of the nuclear envelope (NE) during the mitotic process results in the disassembly of the NPCs into sub-complexes, and about half of the known ones are redirected to other mitotic structures for other relevant functions, to then be reassembled at the end of mitosis [11]. However, despite NUPs, as components of NPCs, having been studied in their main roles in nucleocytoplasmic transport, in maintaining intact overall nuclear architecture, and in transcription regulation, they also participate, with a cooperative or competitive role, in additional functions in the cell-cycle regulation of chromosome segregation during the different mitosis phases, including the spindle assembly checkpoint and kinetochore function with specific targeting of mitotic structures—all relevant processes aimed at the maintenance of genome integrity, including a functional role at the cilium base [12,13,14]. In fact, studies in animal models showed that genetic depletion of NUP transcripts can cause severe defects in zebrafish (loss of the pharyngeal skeletons, small eyes, smaller intestine without folds, and absence of the swim bladder) [15], mice (impairement of neuronal differentiation) [16], and frogs (loss of cilia during embryonic development that can lead to severe congenital heart disease) [14]. Interestingly, some NUPs (such as NUP107, NUP85, NUP133, and NUP160) are expressed in the rostral area and in the intermediate mesoderm of the embryo—critical regions for CNS and pronephric development, respectively [17,18]. Morpholino knockdown of *NUP85* in Xenopus embryos resulted in abnormal pronephros morphology and in glomerulogenesis. Further, homozygous nup85-knockout *Xenopus tropicalis* exhibited a phenotype with small eyes, body axis curvature, edema, and early lethality [17,18]. In previous human studies, various pathogenic *NUP85* variants were described as associated with heart problems and NS [18].

**Figure 1:**
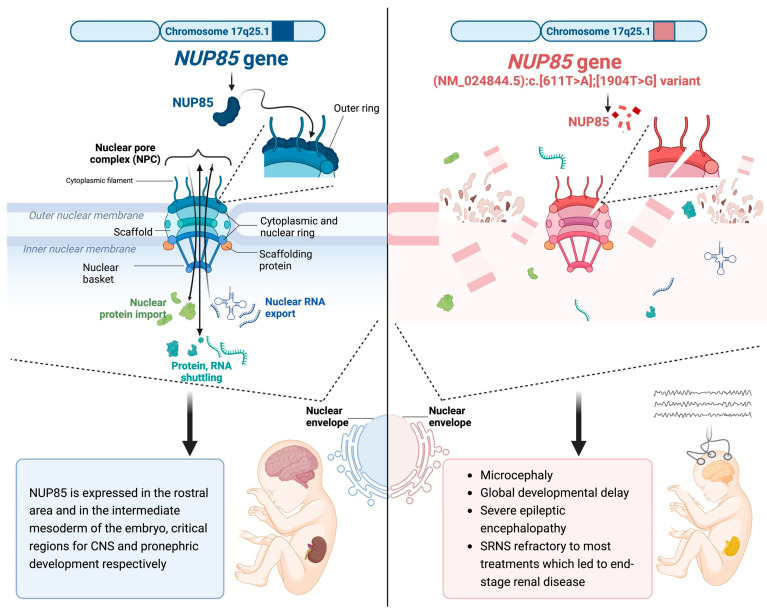
Molecular pathways of NUP85 interactions (created using BioRender.com, accessed on 14 September 2023).

Interestingly, biallelic mutations in *NUP85* have also been associated with a neurodevelopmental disorder characterized by microcephaly, developmental delay, epilepsy, agenesis of the corpus callosum, and dysmorphic features [19]. Specifically, NUP85 was described to act in a manner leading to the proper cilia localization of NUP98 and to modulate both cytoskeletal structures and mitotic machinery in neurons during brain development, like other NUP members (NUP37, NUP107, and NUP214). Indeed, mutations in genes involved in microcephaly pathophysiology mainly affect the mitosis process during proliferation and/or differentiation of neurons (Figure 1).

In this report, we describe an Italian family with an individual affected by microcephaly, global developmental delay, and severe epileptic encephalopathy, who developed an SRNS refractory to most treatments which led to end-stage renal disease. A Whole-Exome Sequencing (WES) showed novel compound heterozygous variants in *NUP85* (NM_024844.5): c.[611T>A]; [1904T>G], inherited from the father and the mother, respectively. We also provide a literature review to compare and expand the clinical spectrum associated with NUP85 pathogenic variants.

## 2. Clinical Report

**Figure 2:**
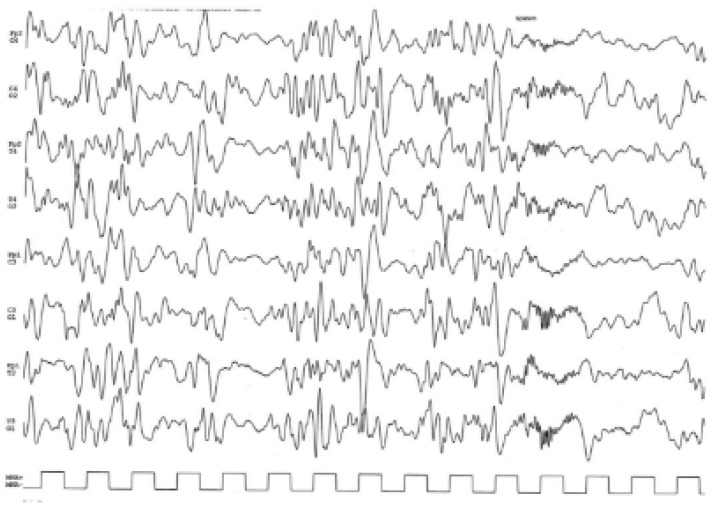
Electroencephalogram (EEG) performed at 4.6 months displays an interictal hypsarrhythmic pattern and ictal EEG (spasm) with a high-voltage slow wave followed by low and fast activity.

**Figure 3:**
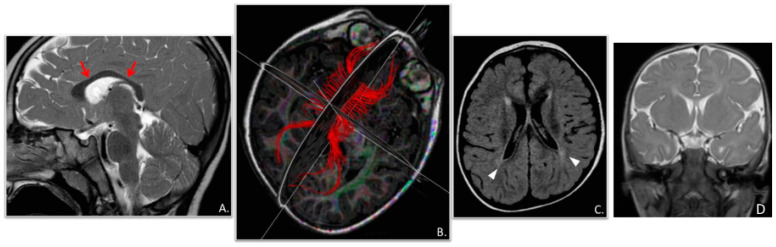
Brain magnetic resonance imaging (MRI) performed at 2.5 years old. (**A**) A midline sagittal T2-weighted Fast Spin-Echo (FSE) image shows mild corpus callosum (CC) hypoplasia with prevalent involvement of the CC trunk (arrows). (**B**) Diffusion Tensor Imaging (DTI) Tractography with CC (red color) fiber tracking. (**C**) An Axial Fluid-Attenuated Inversion Recovery (FLAIR) image displays bilateral paratrigonal white matter hyperintensity (arrowheads). (**D**) Simplified gyration of the cerebral cortex.

**Figure 4:**
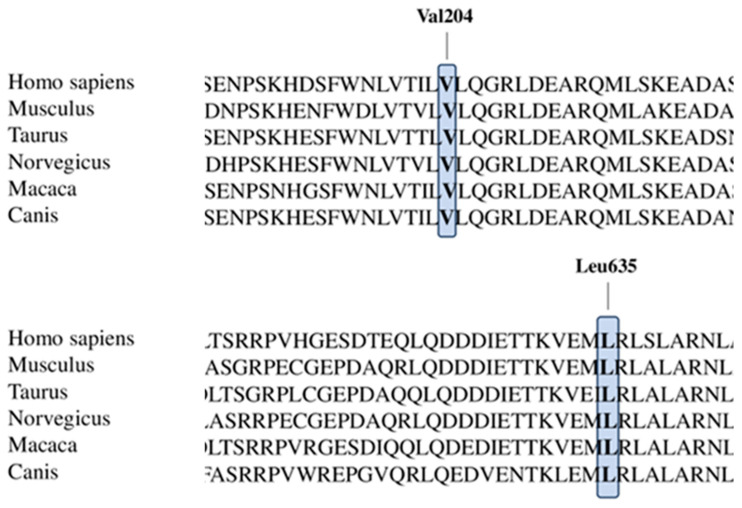
NUP85 protein conservation across species; note the high conservation of mutated residues identified in this study.

Our male patient was the second child born to non-consanguineous and healthy parents. His older sister is unaffected. He was born at 39.5 weeks of gestation after a physiological pregnancy with a birth weight of 2700 g (10th percentile), height of 48 cm (10th percentile), and head circumference of 33 cm (3rd percentile). APGAR scores were 10/10 at 5 and 10 min, respectively. There was no history of hypoxia or asphyxia at birth. Since birth, microcephaly (<3rd centile) and axial hypotonia were noticed. Developmental milestones were severely delayed after the first months of life. At 3 months of age, he showed frequent daily clusters of symmetric epileptic spasms upon awakening, associated with diffuse interictal EEG abnormalities, a hypsarrhythmic pattern, and ictal EEG with a high-voltage slow wave followed by low and fast activity (Figure 2). At their onset, infantile spasms were treated with Vigabatrin and Pyridoxine (with no improvement) and then with adrenocorticotropic hormone, which was partially effective, but this drug was withdrawn due to the onset of arterial hypertension with secondary ventricular hypertrophy. Several drugs (Zonisamide, Topiramate) were ineffective in seizure control. When the child was 2 years old, Valproate (30 mg/kg/day) and Levetiracetam (50 mg/kg/day) were added, resulting in a partial decrease in the frequency and duration of clusters, leading to a seizure-free interval of 3 weeks, associated with the disappearance of the hypsarrhythmic pattern and an improvement in regular background activity. MRI revealed mild hypoplasia of the corpus callosum, mainly in its anterior portion and knee, delayed myelination, and simplified gyration of the cerebral cortex (Figure 3). At his last neurological follow-up at the age of 3 years, medical examination showed hypotonia, reduced or absent osteotendinous reflexes, poor spontaneous motility, and exaggerated reaction to noise. He only achieved inconstant head control in terms of his motor development; the language domain was also severely affected, and he only achieved babbling, but visual interaction was present, although inconstant. At the age of 3 years old, he presented hypoalbuminemia (2.4 g/dL) and proteinuria (urine protein/creatinine ratio of ≥200 mg/g), without edema and with normal serum creatinine. He was referred to the Pediatric Nephrology Department (University of Messina and Bambino Gesù Hospital in Rome, Italy) where he was treated with prednisolone at a dose of 60 mg/m^2^ daily for 8 weeks, without any improvement. Because of persistent hypoalbuminemia, he was started on intravenous albumin at a dose of 10 g every 10 days, while for persistent proteinuria, he was started on Angiotensin Converting Enzyme Inhibitor. A renal biopsy showed focal segmental glomerulosclerosis. A cycle of calcineurin inhibitors (Tacrolimus) was tried, with no response. At 4 years old, he underwent hemodialysis by means of a central venous catheter. He died of sepsis 32 months after the onset of NS. Metabolic screening and karyotype analysis were normal; an NGS panel of 301 genes for epilepsy, neurodevelopmental disorders (NDDs), and cerebral malformations did not reveal abnormalities. WES was performed in the proband and their unaffected parents and displayed a compound heterozygous NUP85 [NM_024844.5]: c.611T>A (p.Val204Glu), c.1904T>G (p.Leu635Arg) variant inherited from the father and mother, respectively. Genomic DNA was isolated from 1 mL of peripheral blood and enriched with SureSelect Clinical research exome 54 Mb (Agilent Technologies, Santa Clara, CA, USA). WES runs were performed on Illumina sequencers, using a standard Illumina pipeline as described before. Paired-end reads were mapped to the reference human genome sequence (GRch37/hg19), after removal of duplicates. The variants were filtered for in-house variant controls and GnomAD databases. Following the pedigree and phenotype, our filtering strategy prioritized rare (<1% in public databases, including the 1000 Genomes project and gnomAD) biallelic and X-linked hemizygous coding variants and/or variants located in genes previously implicated in NDDs and/or epilepsy. Validation, parental origin of the resulting variants, and family segregation studies were performed by means of traditional Sanger sequencing. Both variants are missense mutations and have not been reported in gnomAD, ClinVar, or the 1000 G datasets. Both amino acid residues are strongly conserved through evolution (Figure 4), and the variants are predicted as pathogenic by most computational scores (PolyPhen-2, Mutation Taster, Varsome).

## 3. Discussion

NDDs represent monogenic conditions with genetic heterogeneity, characterized by variable abnormal development of language, motor abilities, cognition, and behavior and frequent neurological comorbidities such as epilepsy and movement disorders [20]. In recent years, NGS technologies, including exome and genome studies, showed an increased complexity underlying NDDs, with frequent involvement of genes implicated in synaptic plasticity/function, chromatin remodelers, regulation of transcription/translation, or the cytoskeleton and cytoskeleton organization [21,22,23,24].

The association between NS and neurological disorders was first described in 1968 and identified as Galloway–Mowat syndrome (MIM 251300). Subsequent reports, however, have shown phenotypic variability and genetic heterogeneity. Colin et al. (2014) [25] reported two patients, from two distinct families, with postnatal microcephaly, severe neurological impairment, and NS associated with a mutation in *WDR73*. This gene encodes a protein containing WD40 repeats, ubiquitously expressed in normal human tissues, including the brain and kidneys, whose function concerns the regulation of the cell cycle, thus playing an important role in brain development and in the maintenance of the proper function and integrity of glomerular filtration [26]. Nevertheless, Colin et al. (2014) [25], expanding the gene sequencing to 26 subjects with microcephaly without NS, did not find *WDR73* mutations, confirming the genetic heterogeneity of the syndrome. Recently, it has been reported that NUPs can play a role in SRNS. Indeed, mutations of *NUP107*, *NUP85*, *NUP133*, and *NUP160*, encoding four components of the outer rings of the NPC, were detected in 29 individuals of 13 families with SRNS [18]. Notably, the study revealed that the *NUP107* c.303G>A (p.Met101Ile) mutation was recurrent, as it has been identified in five families with the combined phenotype, SRNS–microcephaly and ID [18]. Since the same mutation had previously been identified in three additional families with the dual phenotype [27,28], it could be hypothesized that the variant is a hotspot. Homozygous or compound heterozygous mutations in *NUP85* were previously described as the cause of NS type 17 (NPHS17), a subtype of SRNS that inevitably progresses to end-stage renal disease, requiring dialysis or renal transplantation for survival [18]. NPHS17 is a very rare autosomal recessive disease (OMIM*618176) that was previously described only in four patients from three unrelated families who presented, between 4 and 11 years of age, SRNS and microscopic hematuria, progressing to end-stage renal disease. Renal biopsy showed, in all patients, focal segmental glomerulosclerosis [18]. Two families carried different homozygous pathogenic *NUP85* variants associated with SRNS without microcephaly and brain defects, and one carried two compound heterozygous pathogenic *NUP85* variants; notably, two siblings from the latter family showed SRNS and ID without brain abnormalities [18]. These data could suggest that pathogenic *NUP85* variants spare relevant neurological impairment. Recently, however, two individuals with autosomal recessive congenital microcephaly and Seckel syndrome spectrum disorders with neurological impairment have been described, both carrying two different pathogenic variants of *NUP85* (one in homozygosity and the second in compound heterozygosity) without SRNS [19]. Our report describes a novel NPHS17-related compound heterozygous *NUP85* variant associated with microcephaly, severe epileptic encephalopathy, and severe neurological impairment, expanding the phenotype including SRNS and neurologic disease. The severe phenotype of our patient suggests that *NUP85* plays a relevant functional role in the cellular cycle of nephrocyte and neuron progenitors, although we do not know the pathogenic mechanisms of the *NUP85* c.611T>A, 1904T>G variant. However, the factors underlying broader versus narrow phenotypes, such as isolated SRNS or isolated neurological impairment, are still unclear. Therefore, the few data available only suggest that the broader the phenotype, the more severe the dysfunction of the mutated transcript is.

We summarized the clinical phenotypes associated with pathogenic *NUP85* missense variants reported so far in the literature. Seven individuals were examined. The age of onset of clinical features was variable. Congenital microcephaly was present in 3/7 patients (42.8%) in the literature and in our individual.

Moderate intellectual disability (ID) and motor delay were present in 4/7 patients (57.1%) and in our proband. Moreover, two patients (28.5%) showed hypotonia with poor muscle mass, asthenia, and severe speech delay, limited to rudimentary sounds, similarly to our patient. Four individuals (57.1%) displayed short stature and SRNS, while two probands showed facial dysmorphisms. Our individual also presented SRNS and developed an epileptic and developmental encephalopathy with infantile spasms that was refractory to antiseizure medications (ASMs). Two other individuals showed epileptic and developmental encephalopathy with focal onset seizures, refractory to ASMs, as well. Interestingly, only individuals carrying compound heterozygous pathogenic variants (our individual and individuals 1 and 3 in the previous literature) showed cranial MRI abnormalities and structural brain defects, with severe neurodevelopmental impairment and high lethality, suggesting a more severe impact on protein amount and function. Specifically, brain MRI abnormalities included delayed myelination, complete agenesis and/or hypoplasia of the corpus callosum, grey matter heterotopia, cortical malformation of the left frontal lobe, the absence of the cingulate gyrus, and simplified gyration of the cerebral cortex (refer to Table 1 for additional clinical details). These results can be largely explained by functional studies that revealed how the kind of mutation, such as hypomorphic and/or loss of function, led to variable severity phenotypes [29]. Specifically, missense variants affecting conserve residues of NUP85 impaired the protein function and its interaction with substrates, with distinct and organ-specific consequences [18,29]. Missense mutations p.Ala581Pro and p.Arg645Trp and splice site mutation c.405+1G>A of *NUP85* were reported to prevent the interaction between NUP85 protein and its binding partner NUP160 and were associated with SRNS. By contrast, the *NUP85* p.Ala477Val variant might be milder than the others, and it was associated with a residual protein activity [18]. However, the related phenotype was not significantly different from the others. Notably, this residue was not conserved across species, and this mutation presumably impaired the human protein to a greater extent than what occurred in other species. Moreover, the truncating mutation p.Arg107Cysfs*15 of *NUP85* led to a complete loss of function of the protein with early lethality, neurodevelopment impairment, and embryonal death in zebrafish models. Concurrently, mutations affecting the interaction of the NUP85 protein with cytoskeletal proteins and components of mitotic machinery during brain developmental stages led to a neurological phenotype including microcephaly, developmental delay, and epilepsy [19,29]. According to these findings, three types of mutations have been described: complete loss-of-function mutations with embryonal death, not detected in humans; partial loss-of-function mutations mainly affecting brain development and mainly related to a neurological phenotype; and missense hypomorphic mutations leading to specific consequences on protein functions in different organs and tissues [18].

Taken together, these studies confirm the heterogeneity of the dual SRNS–neurological impairment phenotype and, further, the increasing involvement of several NUPs, including *NUP85*, in the pathogenesis of SRNS. Of course, a functional study is the golden standard to prove the pathogenic consequences of a variant. Simultaneously, building a database of future recurrent pathogenic *NUP85* variants associated with defined pathological phenotypes, as reported above concerning the *NUP107* c.303G>A variant, could be a very useful surrogate tool in clinical work.

## Figures and Tables

**Table 1:** Clinical features of individuals with *NUP85* pathogenic variants (adapted by Ravindran et al. [29]).

	Our Study	Ravindran et al., 2023 [29]	Ravindran et al., 2021 [19]		Braun et al., 2018 [18]			
	Our Individual	Individual 1	Individual 2	Individual 3	Individual 4	Individual 5	Individual 6	Individual 7
*NUP85* variant (NM_024844.5)	c.611T>A, c.1904T>G	c.454C>A, c.487C>A	c.932G>A	c.1109A>G, c.1589T>C	c.1430C>T	c.1933C>T	c.405+1G>A	c.1741G>C
Amino acid sequence changes	p.Val204Glu, p.Leu635Arg	p.Leu152Ile, p.Leu163Ile	p.Arg311Gln	p.Asn370Ser, p.Met530Thr	p.Ala477Val	p.Arg645Trp	Donor splice site	p.Ala581Pro
Parents’ consanguinity	−	−	+	−	+	−	−	−
Sex	Male	Male	Female	Female	Female	Male	Female	Male
Age at last assessment	4 years	3.6 years	9 years	27 GW	8 years	11 years	7 years	4 years
Age at onset	birth	birth	birth	prenatal	8 years	11 years	7 years	4 years
Congenital microcephaly	+	+	+	+	NC	NC	NC	NC
Intrauterine growth retardation	−	−	+	−	NC	NC	NC	NC
Short stature	−	+	+	−	+	−	−	+
Underweight	−	−	+		NC	NC	NC	NC
Upslanted palpebral fissures	−	−	+	−	NC	NC	NC	NC
Short philtrum	−	+	+	−	NC	NC	NC	NC
High nasal bridge	−	−	+	−	NC	NC	NC	NC
Reduced vision	−	−	+	Unknown	NC	NC	NC	NC
Optic nerve atrophy	−	−	+	Unknown	NC	NC	NC	NC
Astigmatism	−	+	+	Unknown	NC	NC	NC	NC
Esophoria	−	+	+	Unknown	NC	NC	NC	NC
Long, skinny fingers	−	−		−	NC	NC	NC	NC
Syndactyly	−	−	+	−	NC	NC	NC	NC
Pes adductus	−	+	+	−	NC	NC	NC	NC
Epilepsy	+	−	+	N/A	NC	NC	NC	NC
Intellectual disability, moderate	+	+	+	N/A	−	−	+	+
Delayed speech and language development	+	+	+	N/A	NC	NC	NC	NC
SRNS	+	−	−	N/A	+	+	+	+
Muscular hypotonia	+	+	+	N/A	NC	NC	NC	NC
Cranial MRI abnormalities	+	+	−	+	−	−	−	−

GW, weeks of gestation; MRI, magnetic resonance imaging; SRNS, steroid-resistant nephrotic syndrome; +, yes; –, no; NC, not commented; N/A, not applicable.

## Data Availability

Not applicable.
